# Ethyl Vanillin Protects against Kidney Injury in Diabetic Nephropathy by Inhibiting Oxidative Stress and Apoptosis

**DOI:** 10.1155/2019/2129350

**Published:** 2019-11-12

**Authors:** Yuna Tong, Shan Liu, Rong Gong, Lei Zhong, Xingmei Duan, Yuxuan Zhu

**Affiliations:** ^1^Department of Nephrology, The Third People's Hospital of Chengdu, Chengdu 610031, China; ^2^Department of Laboratory Medicine, Affiliated Hospital of University of Electronic Science and Technology, Sichuan Academy of Medical Sciences and Sichuan Provincial People's Hospital, Chengdu 610072, China; ^3^Personalized Drug Therapy Key Laboratory of Sichuan Province, Sichuan Academy of Medical Science & Sichuan Provincial People's Hospital, University of Electronic Science and Technology of China, Chengdu 610072, China

## Abstract

Diabetes-induced oxidative stress and apoptosis is regarded as a critical role in the pathogenesis of diabetic nephropathy (DN). Treating diabetes-induced kidney damage and renal dysfunction has been thought a promising therapeutic option to attenuate the development and progression of DN. In this study, we investigated the renoprotective effect of ethyl vanillin (EVA), an active analogue of vanillin isolated from vanilla beans, on streptozotocin- (STZ-) induced rat renal injury model and high glucose-induced NRK-52E cell model. The EVA treatment could strongly improve the deterioration of renal function and kidney cell apoptosis *in vivo* and *in vitro*. Moreover, treating with EVA significantly decreased the level of MDA and reactive oxygen species (ROS) and stabilized antioxidant enzyme system in response to oxidative stress by enhancing the activity of superoxide dismutase (SOD), catalase (CAT), and glutathione peroxidase (GSH-Px) *in vivo* and *in vitro*. Furthermore, EVA also markedly suppressed cleaved caspase-3, Bax, and nuclear transcription factor erythroid 2-related factor (Nrf2) expression in STZ-induced rats. Therefore, these results of our investigation provided that EVA might protect against kidney injury in DN by inhibiting oxidative stress and cell apoptosis.

## 1. Introduction

Diabetic nephropathy (DN) is a microvascular complication of diabetes mellitus (DM) and leads to end-stage renal disease [1]. The common clinical and pathological features of DN contain mesangial cell proliferation, glomerular hypertrophy, and thickening of the tubular basal and glomerular membranes, which ultimately develop into fibrosis and chronic renal failure [2, 3]. However, due to a lack of effective pharmacological treatments, DN has accounted for increasing morbidity and mortality in the world. Currently, exogenous insulin was used to regulate blood glucose levels in DN patients. Although it is successful in controlling blood glucose levels, the clinician has to balance between adequate glycemic control and adverse effects related to insulin overdose administration [4, 5]. Recently, although several possible molecular mechanisms underlying DN have been investigated, the specific mechanism of DN remains unidentified. It is reported that hyperglycemia has been considered as an important role in the pathogenesis and development of DN [6, 7].

Several studies indicated that hyperglycemia might promote overproduction of reactive oxygen species (ROS) and oxidative stress, which caused renal fibrosis and induced severe renal injury through bringing out DNA injury, lipid peroxidation, and mitochondrial dysfunction [8, 9]. It is shown that transforming growth factor-*β* (TGF-*β*) has been verified as a potential fibrogenic factor involved in the progression of DN [10]. Furthermore, overproduction of ROS and oxidative stress in DN condition lead to enzyme inactivation, redox imbalance, cell membrane injury, and cell apoptosis [11, 12]. Additionally, excessive ROS induces the mitochondria-dependent apoptotic pathway that implicate in the pathogenesis of DN [13]. Therefore, these studies potently indicated that the prevention of oxidative stress becomes a promising alternative for the therapy of DN. Recently, increasing researches have explored the effect of antioxidant treatments to ameliorate the initiation and progression of DN. Several antioxidant drugs such as taurine, melatonin, vitamin C, and vitamin E are used for treatment and protection of diabetes, but the therapeutic efficacy of these drugs is seriously limited by one or more factors such as adverse effects and inability to regulate blood glucose always [14–16]. Thus, due to the restriction of current therapies, searching for an alternative antidiabetic solution for the treatment of DN remains an important challenge in drug discovery.

**Figure 1:**
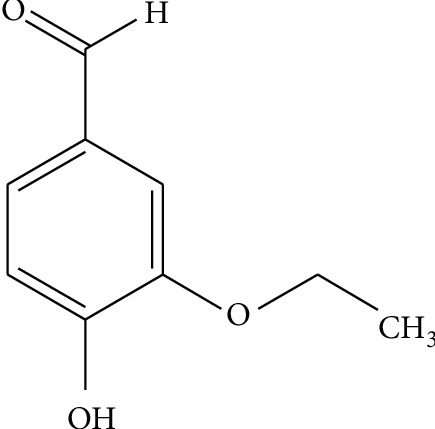
Chemical structure of ethyl vanillin.

Vanillin, a single molecule, is a primary active component extracted from vanilla beans, which has long been used in perfume, food, and medicine [17, 18]. Ethyl vanillin (EVA), an analogue of vanillin, is popularly used as the food additives nowadays (Figure 1). It is reported that LD50 of EVA orally in rats is >3160 mg/kg and LD50 of EVA transdermal in rats is >2000 mg/kg [19]. Because of its safety and long-established use as a food additive, a number of studies have investigated the multifunctional effects of EVA, including antioxidant, antimutagenic, antiangiogenetic, anticolitis, antisickling, and antianalgesic [20, 21]. Recently, several researches suggested that the antioxidant activity of EVA was much stronger than that of vanillin in the oxidative hemolysis inhibition assay [22]. In addition, EVA has an ability against the enhanced ROS level and metalloproteinase-9 in the LPS-stimulated macrophage cells, indicating that it could protect neurodegeneration from oxidative damage [23, 24]. It has been reported that EVA plays a protective role against protein oxidation and apoptosis in rotenone-induced rat model of Parkinson's disease [25]. Furthermore, EVA has the ability to alleviate cell injury induced by CCl_4_ in rats through reducing oxidative stress response [26]. However, the associated scientific literature on the effect of EVA against DN has not been investigated. In our study, the protective effect of EVA against DN has been evaluated using streptozotocin- (STZ-) induced rat model and high glucose-induced cytotoxicity in renal tubular epithelial cells (NRK-52E cells). In addition, the potential mechanism of that activity has also been explored.

## 2. Material and Methods

Ethyl vanillin, streptozotocin (STZ), and D-(+)-glucose powder were obtained from Sigma-Aldrich (MO, USA). Dulbecco's modified Eagle's medium (DMEM) and fetal bovine serum (FBS) were acquired from Gibco (CA, USA). The kits for malondialdehyde (MDA), superoxide dismutase (SOD), catalase (CAT), glutathione peroxidase (GSH-Px), and lactate dehydrogenase (LDH) were obtained from Jiancheng Bioengineering Institute (Nanjing, Jiangsu, China). Reactive oxygen species (ROS) assay kit and nuclear and cytoplasmic protein extraction kit were purchased from Beyotime Institute of Biotechnology (Haimen, Jiangsu, China). Polyclonal antibodies against Bax, cleaved caspase-3, and Nrf2 protein were acquired from Cell Signaling Technology (Danvers, MA, USA). *β*-Actin-specific antibody was provided from Bioss (Beijing, China).

### 2.1. Experimental Animals and Induction of DN

Male Sprague Dawley (SD) rats were obtained from the Experimental Animal Center of Sichuan Provincial People's Hospital. All rats received humane care according to *Guide for the Care and Use of Laboratory Animals*, National Institutes of Health. All protocols and experimental procedures were approved by the Institutional Animal Care and Use Committee of Sichuan Provincial People's Hospital and performed in accordance with the guidelines of the National Act on the use of experimental animals (China).

After an overnight fast, DN rats were induced by injecting a single dose of STZ at the concentration of 60 mg/kg (0.1 M citrate buffer, pH 4.5, i.p.). The normal control rats were administrated with an equivalent amount of citrate buffer. DN rats were confirmed by detecting blood glucose 3 days after STZ administration. The rats with a fasting blood glucose content higher than 16.7 mmol/l were confirmed to have diabetes. The blood glucose level was measured via a strip-operated reflectance meter (Free style K-F095-33749, USA).

The rats were randomly divided into four groups (*n* = 8) for different treatment. The normal control rats (control group) received a single injection of citrate buffer and were treated oral saline solution daily for 8 weeks. The DN rats (STZ group) received a single injection of STZ and were treated oral saline solution daily for 8 weeks. The DN rats treated with 15 mg/kg EVA (STZ+15 mg/kg EVA group) received a single injection of STZ and were treated oral 15 mg/kg of EVA solution daily for 8 weeks. The DN rats treated with 75 mg/kg EVA (STZ+75 mg/kg EVA group) received a single injection of STZ and were treated oral 75 mg/kg of EVA solution daily for 8 weeks. At the end of the 8 weeks, the rats were anesthetized with pentobarbital and sacrificed. The kidneys and blood samples were collected and stored at -80°C for further biochemical evaluation.

### 2.2. Determination of BUN and SCr Contents in the Serum

Serum samples were collected by centrifugation at 3000 rpm for 10 min. The content of blood urea nitrogen (BUN) and serum creatinine (SCr) was detected using an automatic biochemistry analyzer.

### 2.3. Histological Observation and TUNEL Staining

Kidney tissues were fixed in 10% formalin solution for 72 h at 4°C. Then, the samples were routinely processed and embedded in paraffin. Tissue blocks were cut into 4 *μ*m sections using a microtome, and the sections were stained with hematoxylin-eosin (H&E) to observe the histological changes in the renal structure. For TUNEL analysis, the sections were treated with a blocking and permeabilizing solution (0.1% TritonX-100 in 0.1% sodium acetate) for 30 min at 4°C. Then, the sections were stained with the TUNEL reaction solution for 1 h at 37°C, followed by staining with peroxidase-conjugated antibody for 30 min. Then, the sections were treated with diaminobenzidine substrate to produce a dark brown precipitate.

### 2.4. Immunohistochemical Staining

Immunohistochemical staining was used to observe the expression of cleaved caspase-3 and Bax protein in the kidney. Kidney tissues were fixed in 10% formalin solution for 72 h at 4°C. Then, the samples were routinely processed and embedded in paraffin. After that, tissues were cut into 4 *μ*m thickness for immunohistochemical staining. The sections on polylysine-coated slides were incubated with cleaved caspase-3 (1 : 1000) and Bax (1 : 1000) antibodies at 4°C overnight. After rinsing three times with PBS, the sections were treated with a secondary antibody (1 : 500) for 4 h at room temperature. Then, the sections were treated with diaminobenzidine substrate to produce a dark brown precipitate. Assay procedures complied with the manufacturers' instructions. The images were observed using a fluorescence microscope (Carl Zeiss Shanghai Co., Ltd.).

### 2.5. Lipid Peroxidation Assay

Kidney samples were washed with ice saline and homogenized in saline. After homogenization, the solution was centrifuged at 12000 rpm for 10 min at 4°C. The collected supernatants were utilized for analysis of MDA content. The level of MDA, a marker of lipid peroxidation, was measured as thiobarbituric acid reacting substance using a commercial assay kit according to the manufacturer's instruction. The absorbance of solution was measured at 532 nm with a spectrophotometer.

### 2.6. Determination of the Antioxidant Enzyme Activity in the Kidney

Kidney samples were washed with ice saline and homogenized in saline. After homogenization, the solution was centrifuged at 12000 rpm for 10 min at 4°C. The collected supernatants were utilized for the measurement of superoxide dismutase (SOD), catalase (CAT), and glutathione peroxidase (GSH-Px) levels.

The levels of SOD, CAT, and GSH-Px in the kidneys were measured by biochemical methods according to the instructions of the reagent kits. The activities of SOD were determined in terms of xanthine-xanthine oxidase system at 550 nm wavelength using a spectrophotometer. The activities of CAT were assessed by detecting the rate of decomposition of H_2_O_2_ at 405 nm wavelength with a spectrophotometer. The activities of GSH-Px were determined by the DTNB colorimetric assay at 412 nm wavelength using a spectrophotometer. These contents were normalized to the protein concentration in each sample.

### 2.7. Cell Culture

Rat renal tubular epithelial cell (NRK-52E cell) was purchased from the American Type Culture Collection (ATCC, Manassas, VA, USA) and incubated in DMEM supplemented with 10% FBS, 100 U/ml penicillin, and 100 *μ*g/ml streptomycin at 37°C in a humidified atmosphere of 5% CO_2_.

### 2.8. Cell Cytotoxicity Assay

To investigate the protective effect of EVA against DN *in vitro*, cell viability was measured using the MTT assay as in a previous study. Briefly, NRK-52E cells were cultured with normal glucose (NG, 5.6 mM glucose), high glucose (HG, 30 mM glucose), and high glucose with EVA at different concentration (1, 3, and 10 *μ*g/ml) for 48 h. After treatment, 20 *μ*l of MTT (5 mg/ml) was added to each well and incubation continued at 37°C for 4 h according to the manufacturer's instruction. Cell viability was determined at 570 nm using a microplate reader (Bio-Rad, Hercules, CA, USA).

### 2.9. Cell Apoptosis and TUNEL Assay

To further investigate the protective effect of EVA against DN in vitro, cell apoptosis was measured using the Annexin V-PI staining and TUNEL staining as in a previous study. Briefly, NRK-52E cells were cultured with normal glucose (NG, 5.6 mM glucose), high glucose (HG, 30 mM glucose), and high glucose with EVA at different concentration (1, 3, and 10 *μ*g/ml) for 48 h. After treatment, NRK-52E cells were harvested with 0.25% trypsin, washed three times with PBS, resuspended in buffer, and cultured with 5 *μ*l Annexin V-FITC and 10 *μ*l propidium iodide for 10 min in the dark at room temperature. Samples were then analyzed by flow cytometry (Beckman Coulter). The degrees of DNA nick formation and genomic DNA fragmentation were observed by TUNEL assay. The cells cultured on coverslips were performed using a TUNEL assay kit according to the manufacturer's instruction. Subsequently, the cells were visualized by a fluorescent microscope (Carl Zeiss Shanghai Co., Ltd). Image analysis of TUNEL staining was performed using ImageJ (NIH, USA).

### 2.10. Intracellular Reactive Oxygen Species Analysis

To monitor the intracellular ROS level, NRK-52E cells were probed with the redox sensitive dye 2′,7′-dichlorodihydrofluorescein diacetate (DCFH-DA). The production of ROS was labeled with green fluorescence and observed by photograph using a fluorescence microscope (Carl Zeiss Shanghai Co., Ltd).

### 2.11. Antioxidant System Assay

The activities of antioxidant enzymes in NRK-52E cells, including SOD, CAT, and GSH-Px, were evaluated by commercial assay kits according to the manufacturer's protocols. The absorbance of samples was detected at 550 nm for SOD, 405 nm for CAT, and 412 nm for GSH-PX at the end of reaction using a microplate reader (Bio-Rad, Hercules, CA, USA).

### 2.12. Western Blotting

Western blotting analysis was used to detect the content of Nrf-2 protein in the kidney and NRK-52E cells. Kidney samples were washed with ice saline and homogenized in saline. After homogenization, the solution was centrifuged at 12000 rpm for 10 min at 4°C to collect the proteins in the supernatant. The supernatant was mixed with loading buffer and boiled for 10 min. The mixed solution was electrophoresed on a 10% SDS-polyacrylamide gel, then the proteins were transferred from the gel on PVDF membrane. The membrane was blocked with 5% nonfat milk in Tris-buffered saline containing 0.1% Tween-20 for 1 h and then incubated with Nrf-2 (1 : 1000) and *β*-actin (1 : 500) antibodies at 4°C overnight. After being washed three times with PBS, the membranes were incubated with peroxidase-conjugated secondary antibody (goat anti-rabbit, 1 : 1000) 1 h and rinsed with TBST buffer three times again.

Western blotting assay was used to evaluate the levels of nuclear-localized Nrf2 protein (n-Nrf2). Briefly, NRK-52E cells were seeded in culture dishes (4 × 10^6^) and incubated with EVA and HG as described for the cell viability assays. The cells were washed with PBS three times and treated with 1 ml of RIPA lysis buffer. Nuclear protein was extracted by using the nuclear and cytoplasmic protein extraction kit according to the manufacturer's instruction. The extracted proteins were detected using western blotting as previously described. Primary antibodies of Nrf-2 (1 : 1000) and Lamin B (1 : 1000) were used in the study. Specific bands were treated for chemiluminescence and visualized using a Bio-Rad Gel Doc XR+ Molecular Imager. The grayscale intensity of bands was quantitated using Gel EQ Quantity One software (Bio-Rad).

### 2.13. Statistical Analysis

The results were represented as mean ± SEM. Differences between groups were compared using one-way ANOVA followed by Dunnett's post hoc test (version 17.0 software, SPSS Inc.). Differences with a *P* < 0.05 were considered to be statistically significant.

## 3. Results

### 3.1. Effect of EVA on Renal Function in STZ-Induced Rats

**Figure 2:**
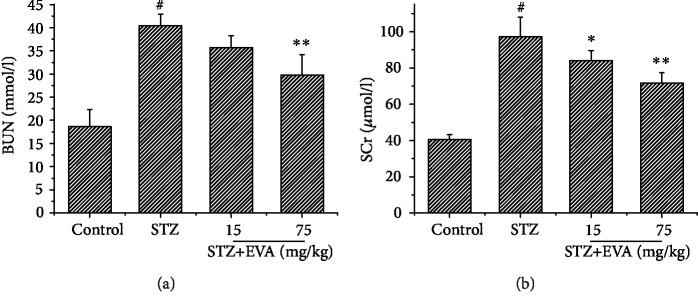
Effect of EVA on the biochemical parameters in STZ-induced rats. EVA was administrated to STZ-induced rats for 8 weeks. (a) Level of BUN was detected by a standard method; (b) level of SCr was detected by a standard method. Results are presented with means ± SEM (*n* = 8). ^#^*P* < 0.01 compared to control group; ^∗^*P* < 0.05 compared to STZ-induced group; ^∗∗^*P* < 0.01 compared to STZ-induced group.

The effect of EVA in protecting renal function was investigated using a STZ-induced SD rat model. After administration with STZ for 8 weeks, the levels of BUN (Figure 2(a)) and SCr (Figure 2(b)) were increased to 40.43 mmol/l and 90.21 *μ*mol/l in diabetic rats, respectively. However, treatment with different concentrations of EVA (15 mg/kg and 75 mg/kg) significantly ameliorated the STZ-induced changes of BUN (35.71 and 25.78 mmol/l, respectively) and SCr (83.95 and 71.67 *μ*mol/l, respectively) in STZ-induced rats. Hence, our results suggested that EVA could ameliorate hyperglycemia and improve the renal function in STZ-treated rats.

### 3.2. Effect of EVA on Renal Impairment and Cell Apoptosis

**Figure 3:**
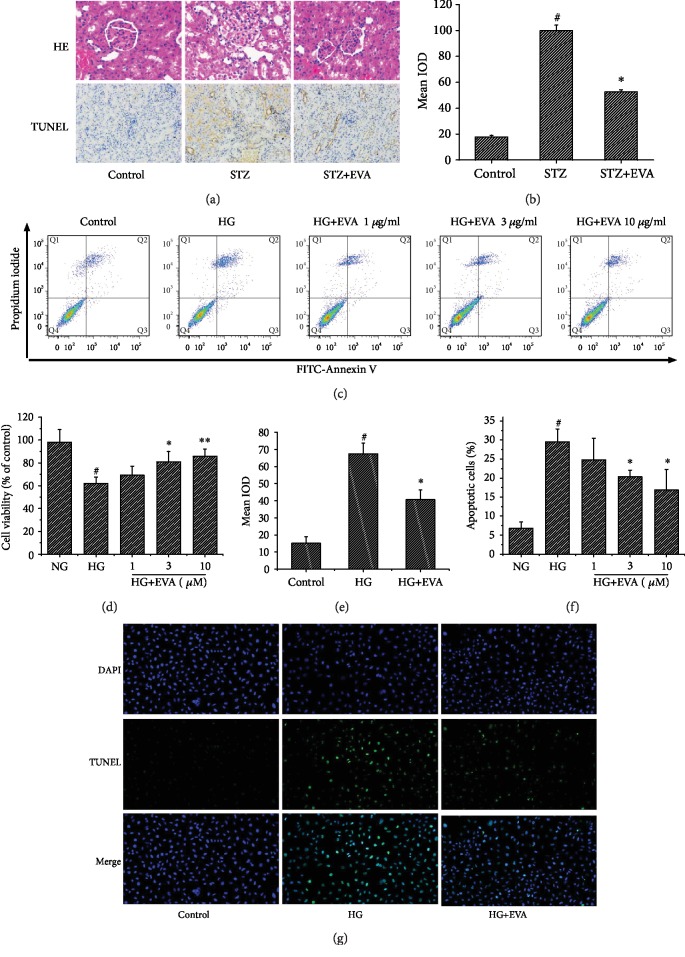
Effect of EVA to protect DN-induced renal injury *in vivo* and *in vitro*. (a) Kidney sections stained with HE and TUNEL staining (×200). (b) Semiquantitative analysis of TUNEL staining in the rat kidney images was shown. Results are presented with means ± SEM (*n* = 8). ^#^*P* < 0.01 compared to control group; ^∗^*P* < 0.01 compared to STZ-induced group. (c) Annexin V/FITC-PI staining and flow cytometric analysis of apoptosis. (d) Cell viability was measured by the MTT assay. Results are presented with means ± SEM (*n* = 5). ^#^*P* < 0.01 compared to control group; ^∗^*P* < 0.05 compared to HG-induced group; ^∗∗^*P* < 0.01 compared to HG-induced group. (e) Semiquantitative analysis of TUNEL staining in the NRK-52E cells was shown. Results are presented with means ± SEM (*n* = 5). ^#^*P* < 0.01 compared to control group; ^∗^*P* < 0.01 compared to HG-induced group. (f) Apoptotic rates of NRK-52E cells in Annexin V/FITC-PI staining were shown. Results are presented with means ± SEM (*n* = 5). ^#^*P* < 0.01 compared to control group; ^∗^*P* < 0.01 compared to HG-induced group. (g) The level of apoptosis in NRK-52E cells analyzed with TUNEL staining (×200).

The protective effect of EVA against STZ-induced renal injury was observed via HE staining and TUNEL assay (Figures 3(a) and 3(b)). It indicated that normal architecture was observed in the control group. After treating rats with STZ for 8 weeks, there are noticeable changes in the renal structures, characterized by glomerular sclerosis and necrosis, degeneration in the tubule epithelium, and tubular dilation. However, these changes were attenuated by treatment with 75 mg/kg EVA. Furthermore, renal cell apoptosis was observed in rats using a TUNEL staining. Treating rats with STZ significantly increases the degree of apoptosis compared with the control group. Interestingly, the level of TUNEL-positive cell was ameliorated slightly by the treatment with 75 mg/kg EVA.

NRK-52E cell viability was detected using an MTT assay (Figure 3(d)). When cells were incubated with HG for 48 h, cell viability was observed to decrease to 62.04% as compared with the control group. However, after being treated with different concentrations of EVA (1, 3, and 10 *μ*g/ml) in NRK-52E cells, cell viability was significantly increased to 69.14, 80.91, and 85.81% of the control group, respectively.

The effect of EVA on HG-induced apoptosis in NRK-52E cells was observed via Annexin V-PI (Figures 3(c) and 3(f)) staining and TUNEL assay (Figures 3(e) and 3(g)). When cells were cultured with HG for 48 h, apoptotic rates of NRK-52E cells were increased to 29.55%. However, after being treated with different concentrations of EVA (1, 3, and 10 *μ*g/ml) in NRK-52E cells, the apoptotic rates were significantly decreased to 24.81, 20.32, and 16.86%, respectively. In the TUNEL assay, treating NRK-52E cells with HG significantly increased the degree of apoptosis compared to the control group. However, this change was reduced by the treatment with 10 *μ*g/ml EVA. The above results suggested that EVA had a promising capacity on improving STZ- or HG-induced renal damage and cell apoptosis both *in vivo* and *in vitro*.

### 3.3. Effect of EVA on Lipid Peroxidation and Oxidative Stress

**Figure 4:**
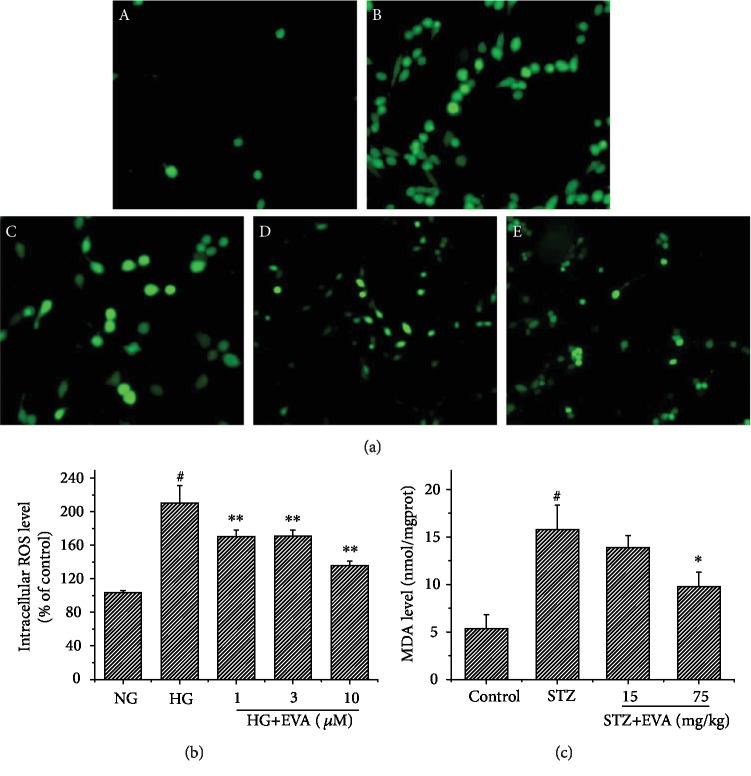
Effect of EVA to protect against diabetes-induced oxidative stress *in vivo* and *in vitro*. (a) Level of ROS was observed by a fluorescence microscope (×400): (A) Control, (B) HG only, (C) HG+1 *μ*M EVA, (D) HG+3 *μ*M EVA, and (E) HG+10 *μ*M EVA. (b) Semiquantitative image analysis of ROS level. Data are presented as the percentage of control group (mean ± SD; *n* = 5). ^#^*P* < 0.01 compared to control group; ^∗∗^*P* < 0.01 compared to HG-induced group. (c) Levels of MDA were detected by a standard method. Results are presented with means ± SEM (*n* = 8). ^#^*P* < 0.01 compared to control group; ^∗^*P* < 0.05 compared to STZ-induced group.

To clarify the role of EVA treatment on lipid peroxidation in STZ-induced rats, the MDA levels in the kidney were detected (Figure 4(c)). The content of MDA was greatly increased to 15.79 nmol/mgprot in STZ-induced rats. In contrast, the levels of MDA (13.88, 9.78 nmol/mgprot, respectively) were decreased in response to a different dose of EVA (15, 75 mg/kg) treatment. Furthermore, to explore the potential ability of EVA on protection of oxidative stress in diabetic nephropathy, we detected the production of ROS in HG-induced NRK-52E cells (Figures 4(a) and 4(b)). After exposure of NRK-52E cells to HG for 48 h, the level of ROS was increased to 210.11% of the control group. While the NRK-52E cells were treated with different concentrations of EVA (1, 3, and 10 *μ*g/ml), the contents of ROS (170.32, 170.79, and 135.81% of the control group, respectively) were significantly attenuated. Therefore, the results suggested that EVA might protect STZ-induced rats and HG-induced NRK-52E cells against renal damage by inhibiting oxidative stress.

### 3.4. Effect of EVA on Antioxidant Enzyme Systems

**Figure 5:**
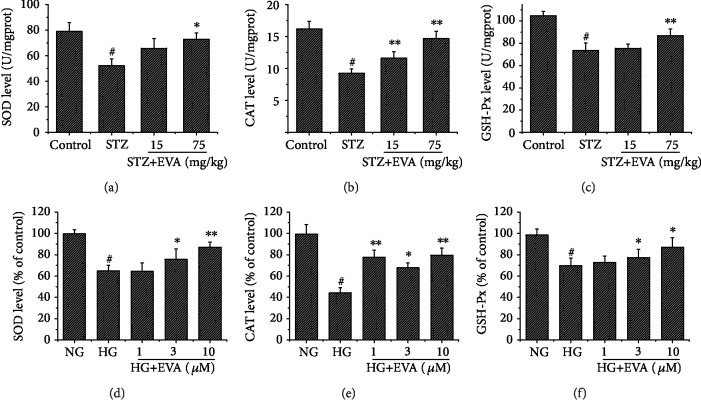
Effect of EVA on antioxidant enzyme activity *in vivo* and *in vitro*. After treatment of EVA on STZ-induced rats, (a) SOD activity was detected in the kidney of DN rats; (b) CAT activity was detected in the kidney of DN rats; (c) GSH-Px activity was detected in the kidney of DN rats. Results are presented with means ± SEM (*n* = 8). ^#^*P* < 0.01 compared to control group, ^∗^*P* < 0.05 compared to STZ-induced group, ^∗∗^*P* < 0.01 compared to STZ-induced group. After treatment of EVA on HG-induced cells, (d) SOD activity was detected using a standard assay; (e) CAT activity was detected using a standard assay; (f) GSH-Px activity was detected using a standard assay. Data are shown as the percentage of the control group, and results are presented with means ± SEM (*n* = 5). ^#^*P* < 0.01 compared to control group, ^∗^*P* < 0.05 compared to HG-induced group, ^∗∗^*P* < 0.01 compared to HG-induced group.

We investigated the effect of EVA on the activity of renal antioxidant enzyme systems such as SOD (Figure 5(a)), CAT (Figure 5(b)), and GSH-Px (Figure 5(c)) in STZ-induced rats. The activities of SOD, CAT, and GSH-Px were greatly diminished to 50.11 U/mgprot, 9.23 U/mgprot, and 73.75 U/mgprot in the renal tissues of STZ-induced rats, respectively. Conversely, treatment with different concentrations of EVA (15, 75 mg/kg) obviously improved the altered activities of SOD (65.69 and 72.83 U/mgprot, respectively), CAT (11.61 and 14.64 U/mgprot, respectively), and GSH-Px (75.53 and 86.91 U/mgprot, respectively) in renal tissues of STZ-induced rats. Moreover, to investigate the protective effect of EVA on antioxidant enzyme systems *in vitro*, we assessed the level of SOD (Figure 5(d)), CAT (Figure 5(e)), and GSH-Px (Figure 5(f)) in HG-induced NRK-52E cells. After exposure of NRK-52E cells to HG for 48 h, the activities of SOD, CAT, and GSH-Px were decreased to 64.71%, 44.16%, and 69.74% compared to the control group, respectively. While the NRK-52E cells were cultured with different concentrations of EVA (1, 3, and 10 *μ*g/ml), the activities of SOD (64.41, 75.62, and 87.06% compared to the control group, respectively), CAT (77.62, 67.66, and 79.52% compared to the control group, respectively), and GSH-Px (72.65, 77.41, and 86.99% compared to the control group, respectively) were significantly improved. Thus, the above results demonstrated that EVA had a potential capacity to ameliorate renal injury via enhancing antioxidant enzyme systems *in vitro* and *in vivo.*

### 3.5. Effect of EVA on the Expression of Nrf2 Protein in STZ-Induced Rats

**Figure 6:**
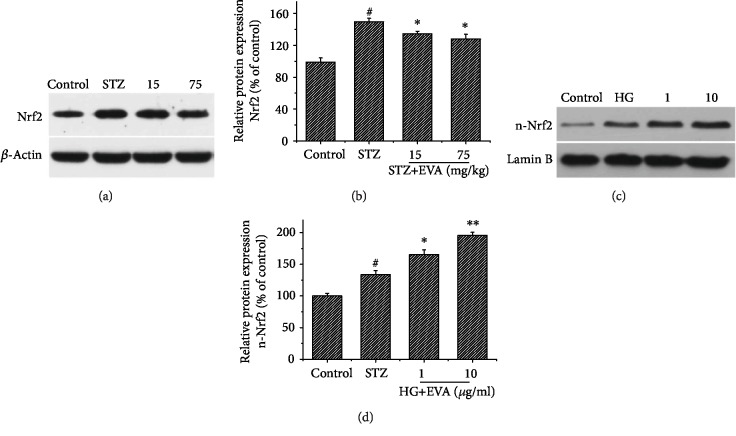
Effects of EVA on the expression of Nrf2 in STZ-induced rats and n-Nrf2 in HG-induced NRK-52E cells. (a) The expression of Nrf2 protein was observed by western blotting. (b) The quantitative analysis of Nrf2 protein expression. Results are presented with means ± SEM (*n* = 3). ^#^*P* < 0.01 compared to control group, ^∗^*P* < 0.05 compared to STZ-induced group. (c) The expression of n-Nrf2 protein was observed by western blotting. (d) The quantitative analysis of n-Nrf2 protein expression. Results are presented with means ± SEM (*n* = 3). ^#^*P* < 0.05 compared to control group, ^∗^*P* < 0.05 and ^∗∗^*P* < 0.01 compared to STZ-induced group.

The capabilities of EVA on protecting against oxidative stress were further explored in the expression levels of oxidative stress-related proteins of Nrf2 (Figures 6(a) and 6(b)). The expression of Nrf2 proteins was significantly increased to 149.66% in STZ-induced rats compared to the control group. However, the different doses of EVA (15 and 75 mg/kg) treatment significantly reduced the expression levels of Nrf2 to 134.44% and 127.88% compared to the control group, respectively. Therefore, this result suggested that EVA was a potent inhibitor of oxidative stress, which might be related to the effect on Nrf2 pathways.

To further explore the effect of EVA on the n-Nrf2 protein in HG-induced NRK-52E cells, the levels of n-Nrf2 protein in NRK-52E cells treated with NG or HG and EVA were confirmed by the western blot analysis (Figures 6(c) and 6(d)). Exposing the NRK-52E cells to HG for 48 h enhanced the level of n-Nrf2. However, the protein content of n-Nrf2 further increased in response to EVA treatment. Collectively, these data suggested that EVA was a potent inhibitor of oxidative stress, which might be related to the activation of Nrf2-mediated defensive system.

### 3.6. Effect of EVA on the Expression of Bax and Cleaved Caspase-3 in STZ-Induced Rats

**Figure 7:**
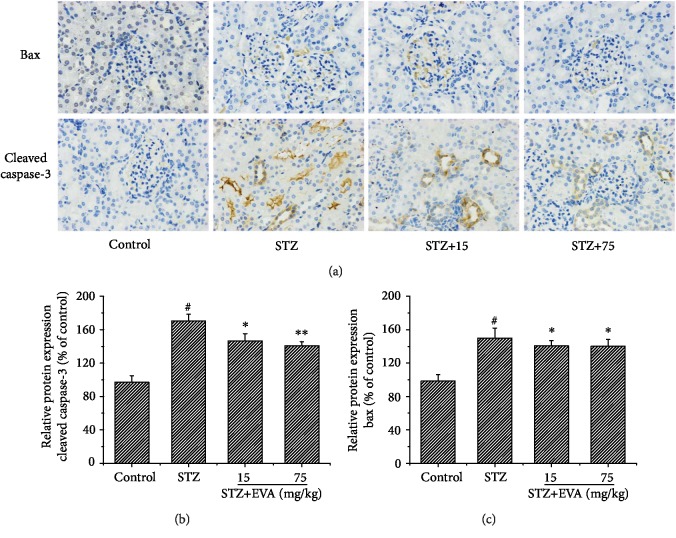
Inhibitory effect of EVA on the changes in Bax and activated caspase-3 in STZ-induced rats. (a) The expression of Bax and cleaved caspase-3 protein was observed by western blotting. (b, c) Semiquantitative analysis of cleaved caspase-3 and Bax protein expression. Results are presented with means ± SEM (*n* = 5). ^#^*P* < 0.01 compared to control group, ^∗^*P* < 0.05 compared to STZ-induced group, ^∗∗^*P* < 0.01 compared to STZ-induced group.

Due to the fact that overproduction of ROS is responsible for renal apoptosis, we observed the expression of apoptosis-related proteins Bax and cleaved caspase-3 using an immunohistochemical staining (Figure 7(a)). The expression of Bax (Figure 7(b)) and cleaved caspase-3 (Figure 7(c)) proteins was greatly increased in STZ-induced rats compared to the control group, indicating more severe renal apoptosis. Promisingly, the treatment of EVA significantly reversed the expression of Bax and cleaved caspase-3 protein as compared to the STZ-induced rats.

## 4. Discussion

The purpose of this study is to investigate the protective effect of EVA against DN both *in vivo* and *in vitro*. Although the precise mechanism of DN has not been completely elucidated, it is reported that the effects of oxidative stress and apoptosis play an important factor in the pathophysiology of DN [27]. Recently, increasing amount of antioxidant and free radical scavenger was used for the treatment of DN. To explore the clinical potential of EVA, we assessed the protective effect of EVA on renal damage using STZ-induced rats and HG-induced NRK-52E cells [28, 29]. Our results suggested that EVA might be a potential protective compound against renal oxidative stress and apoptosis in DN disease.

STZ is widely used to induce experimental renal injury, as it is specifically destroying the islets of Langerhans [28]. Under DN condition, renal functions are degenerated due to injury in the kidney. Some investigators reported that the alteration of several biochemical parameters in the blood played an important role in the progression of DN [30, 31]. Therefore, the levels of BUN and SCr were tested in STZ-induced rats. In the present study, after STZ injection, the rats showed typical renal dysfunction of DN such as increased BUN and SCr level, which is consistent with previous experimental evidences [30]. However, it was observed that treatment with EVA effectively reversed these alterations in renal function by decreasing the level of BUN and SCr, indicating the renoprotective effect of EVA against STZ-induced renal dysfunction. Furthermore, this phenomenon was manifested by the observation of a pathological analysis, as verified by an improvement in the development and progression of renal histopathological lesions.

Previous studies suggested that renal cell apoptosis is also an important progression of DN [27, 29]. According to our result, administrating rats with STZ induced a great enhancement in TUNEL-positive cells in the kidney compared to the control group. However, it was observed that treatment with EVA obviously ameliorated this increase in STZ-induced renal tissue. Additionally, in accordance with *in vivo* experimental evidences, we also observed HG-induced apoptosis directly in NRK-52E cells. Treating cells with EVA could effectively reduce these alterations induced by HG in NRK-52E cells. These results suggested that EVA had an ability to mitigate diabetes-mediated renal apoptosis induced by STZ and HG.

Although the precise mechanism of diabetes nephrotoxicity is still unclear, several opinions from recent investigation strongly indicated that oxidative stress induced by hyperglycemia might be involved in the pathogenesis and development of diabetic nephropathy [32]. Additionally, it was reported that hyperglycemia could result in oxidative injury by triggering oxidative stress, leading to the overproduction of ROS [33]. Kidney cell is more sensitive to ROS associated with oxidative injury due to a high rate of oxygen consumption. Furthermore, excess production of ROS could induce an enhancement in MDA content, an indicator to detect the degree of lipid peroxidation [34]. In the present study, we observed that the rats injected with STZ have an increased level of MDA, which is in accordance with previous studies [30]. At the same time, we also found that the production of ROS in NRK-52E cells was increased significantly after being induced with HG. However, treatment with EVA could attenuate these changes *in vivo* and *in vitro*. Thus, our results indicated that EVA had the ability to antagonize diabetes-induced ROS overproduction and oxidative stress in STZ-induced rats and HG-induced NRK-52E cells.

There is a balance between the production of ROS and the endogenous cellular antioxidant system, including enzymatic and nonenzymatic, under physiological condition. However, more and more animal experiments and clinical investigations indicated that diabetes not only induced the overproduction of ROS but also might interfere the antioxidant defense system via regulating the activities of antioxidant enzyme [35]. Thus, to further prove the renoprotective effect of EVA, we explored the effect of EVA on the level of SOD, CAT, and GSH-Px, important antioxidases in the antioxidant defense system in renal damage, *in vivo* and *in vitro*. It is well known that SOD is a cytoprotective antioxidant defense enzyme, which has the ability to catalyse the dismutation reaction in superoxide radical conversion to hydrogen peroxides and molecular oxygen [36]. Thereafter, CAT and GSH-Px further resolve hydrogen peroxides produced by the dismutation reaction [37, 38]. CAT, a hemeprotein, inhibits the generation of hydroxyl radicals and averts the cellular constituents from peroxisome-mediated oxidative injury [37]. In addition, GSH-Px is a selenium-containing enzyme that detoxifies the lipid hydroperoxides and free hydrogen to water through decreased glutathione oxidation [38]. Therefore, in our study, the activities of SOD, CAT, and GSH-Px in the renal function of DN rats and NRK-52E cells were detected. According to our results, we found that treatment with EVA might ameliorate STZ-induced oxidative injury in DN rats and HG-induced oxidative injury in NRK-52E cells through stabilizing the activities of antioxidant enzyme system.

Currently, several researches verify the effect of the kelch-like ECH-associated protein-1 (KEAP1)/nuclear transcription factor erythroid 2-related factor (Nrf2) system in this process, and the proteins have important effects in cellular protection against oxidative stress [39, 40]. Nrf2 plays a central defensive function in diabetic nephropathy because of its ability of attenuating ROS and regulating redox balance under oxidative stress [41–43]. Overproduction of ROS can activate Nrf2 through releasing it from Keap1 or activating its phosphorylation by several kinases [44]. Then, the activated Nrf2 is entered into the nucleus and interacts with antioxidant response element to promote the transcription of downstream target genes coding for cytoprotective proteins, such as GSH-Px, CAT, and SOD, for antioxidation and detoxication [45, 46], which is consistent with our previous studies. Therefore, Nrf2 has been thought as a potential therapeutic target for preventing and reversing the progression of DN. In our results, we found that the protein expression of Nrf2 in renal function was decreased in STZ-induced rats. However, EVA treatment could inhibit the decrease of Nrf2 expression in renal function. Furthermore, according to our results, EVA treatment obviously improves the NRF2 activation in the nucleus of HG-induced NRK-52E cells, which indicated that the protective effect of EVA in DN might be associated with the Nrf2 pathway.

It is generally accepted that the production of ROS not only induces cytotoxicity but also stimulates the signaling molecules to trigger apoptosis protein expression, including Bax and cleaved caspase-3, eventually leading to the injury and apoptosis of kidney cells [47]. Additionally, several researches confirmed that diabetes could trigger renal intrinsic apoptosis pathways [48]. Therefore, suppression of apoptotic protein expression may be an important factor in the development and progression of DN. Moreover, the proteins Bax and cleaved caspase-3 had been thought as indicator of ROS-induced apoptosis. To deeply explore the potential protective mechanism of EVA against renal apoptosis in STZ-induced rats, the expressions of Bax and cleaved caspase-3 were evaluated. According to our results, Bax and cleaved caspase-3 protein expression was markedly increased in STZ-induced rats. However, administration of EVA inhibited the expression of Bax and cleaved caspase-3 in renal function of STZ-induced rats. The above results suggested that EVA obviously mitigated ROS-mediated renal damage induced by STZ and this protective effect might be associated with the inhibition of the apoptotic pathway.

## 5. Conclusion

**Figure 8:**
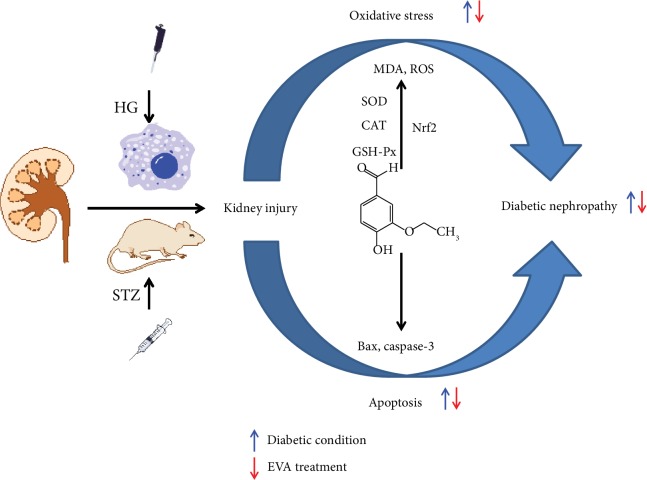
Schematic flow diagram of the renoprotective effects of EVA on HG- and STZ-induced kidney injury and its underlying mechanism.

Taken together, our *in vivo* and *in vitro* studies demonstrated that EVA could protect against diabetic nephropathy through inhibition of oxidative stress and apoptosis and the protective effect might be associated with the regulation of the activation of Nrf2. Therefore, according to our research, EVA may be a promising therapeutic option for preventing DN (Figure 8). Furthermore, more potential mechanisms need to be explored relating to the nephroprotective effect of EVA in the future.

## Data Availability

All the data used to support the findings of this study are included within the article.

## References

[1] Reutens A. T., Atkins R. C. (2011). Epidemiology of diabetic nephropathy. *Contributions to Nephrology*.

[2] Maiese K. (2008). Diabetic stress: new triumphs and challenges to maintain vascular longevity. *Expert Review of Cardiovascular Therapy*.

[3] Hosseini A., Sharifzadeh M., Rezayat S. M. (2010). Benefit of magnesium-25 carrying porphyrin-fullerene nanoparticles in experimental diabetic neuropathy. *International Journal of Nanomedicine*.

[4] Hendig D., Tarnow L., Kuhn J., Kleesiek K., Götting C. (2008). Identification of a xylosyltransferase II gene haplotype marker for diabetic nephropathy in type 1 diabetes. *Clinica Chimica Acta*.

[5] Zhang M., Feng L., Gu J. (2014). The attenuation of Moutan Cortex on oxidative stress for renal injury in AGEs-induced mesangial cell dysfunction and streptozotocin-induced diabetic nephropathy rats. *Oxidative Medicine and Cellular Longevity*.

[6] Hasegawa K., Wakino S., Simic P. (2013). Renal tubular Sirt1 attenuates diabetic albuminuria by epigenetically suppressing Claudin-1 overexpression in podocytes. *Nature Medicine*.

[7] Ruggenenti P., Cravedi P., Remuzzi G. (2010). The RAAS in the pathogenesis and treatment of diabetic nephropathy. *Nature Reviews Nephrology*.

[8] Heyman S. N., Rosen S., Khamaisi M., Idée J. M., Rosenberger C. (2010). Reactive oxygen species and the pathogenesis of radiocontrast-induced nephropathy. *Investigative Radiology*.

[9] Meinel F. G., de Cecco C. N., Schoepf U. J., Katzberg R. (2014). Contrast-induced acute kidney injury: definition, epidemiology, and outcome. *BioMed Research International*.

[10] Deshpande S. D., Putta S., Wang M. (2013). Transforming growth factor-*β*-induced cross talk between p53 and a microRNA in the pathogenesis of diabetic nephropathy. *Diabetes*.

[11] Williams M. E. (2004). Clinical studies of advanced glycation end product inhibitors and diabetic kidney disease. *Current Diabetes Reports*.

[12] Singh D. K., Winocour P., Farrington K. (2011). Oxidative stress in early diabetic nephropathy: fueling the fire. *Nature Reviews Endocrinology*.

[13] Brezniceanu M. L., Lau C. J., Godin N. (2010). Reactive oxygen species promote caspase-12 expression and tubular apoptosis in diabetic nephropathy. *Journal of the American Society of Nephrology*.

[14] Gao Y., Zhang R. R., Li J. H. (2012). Radix Astragali lowers kidney oxidative stress in diabetic rats treated with insulin. *Endocrine*.

[15] Rains J. L., Jain S. K. (2011). Oxidative stress, insulin signaling, and diabetes. *Free Radical Biology & Medicine*.

[16] Antonio C., Roberto T. (2009). Antioxidant anti-inflammatory treatment in type 2 diabetes. *Diabetes Care*.

[17] Wu S. L., Chen J. C., Li C. C., Lo H. Y., Ho T. Y., Hsiang C. Y. (2009). Vanillin improves and prevents trinitrobenzene sulfonic acid-induced colitis in mice. *Journal of Pharmacology and Experimental Therapeutics*.

[18] Lirdprapamongkol K., Sakurai H., Kawasaki N. (2005). Vanillin suppresses in vitro invasion and in vivo metastasis of mouse breast cancer cells. *European Journal of Pharmaceutical Sciences*.

[19] Hagan E. C., Hansen W. H., Fitzhugh O. G. (1967). Lebensmittel-Geschmacksstoffe und Verbindungen verwandter Struktur. II. Subakute und chronische Toxizitat. *Food and Cosmetics Toxicology*.

[20] Makni M., Chtourou Y., Fetoui H., Garoui E. M., Boudawara T., Zeghal N. (2011). Evaluation of the antioxidant, anti-inflammatory and hepatoprotective properties of vanillin in carbon tetrachloride-treated rats. *European Journal of Pharmacology*.

[21] Tai A., Sawano T., Yazama F., Ito H. (2011). Evaluation of antioxidant activity of vanillin by using multiple antioxidant assays. *Biochimica et Biophysica Acta (BBA) - General Subjects*.

[22] Tai A., Sawano T., Yazama F. (2011). Antioxidant properties of ethyl vanillin in vitro and in vivo. *Bioscience, Biotechnology, and Biochemistry*.

[23] Chen X. M., Wei M., Zhang H. M., Luo C. H., Chen Y. K., Chen Y. (2012). Effect of vanillin and ethyl vanillin on cytochrome P450 activity in vitro and in vivo. *Food and Chemical Toxicology*.

[24] Kim J. H., Lee H. O., Cho Y. J. (2014). A vanillin derivative causes mitochondrial dysfunction and triggers oxidative stress in Cryptococcus neoformans. *PLoS One*.

[25] Dhanalakshmi C., Janakiraman U., Manivasagam T. (2016). Vanillin attenuated behavioural impairments, neurochemical deficts, oxidative stress and apoptosis against rotenone induced rat model of Parkinson’s disease. *Neurochemical Research*.

[26] Makni M., Chtourou Y., Barkallah M., Fetoui H. (2012). Protective effect of vanillin against carbon tetrachloride (CCl_4_)-induced oxidative brain injury in rats. *Toxicology and Industrial Health*.

[27] Wagener F. A. D. T. G., Dekker D., Berden J. H., Scharstuhl A., van der Vlag J. (2009). The role of reactive oxygen species in apoptosis of the diabetic kidney. *Apoptosis*.

[28] Brahmanaidu P., Uddandrao V. V. S., Sasikumar V. (2017). Reversal of endothelial dysfunction in aorta of streptozotocin-nicotinamide-induced type-2 diabetic rats by S-Allylcysteine. *Molecular and Cellular Biochemistry*.

[29] Tong Y., Chuan J., Bai L. (2018). The protective effect of shikonin on renal tubular epithelial cell injury induced by high glucose. *Biomedicine & Pharmacotherapy*.

[30] Ma S. T., Liu D. L., Deng J. J., Niu R., Liu R. B. (2013). Effect of arctiin on glomerular filtration barrier damage in STZ‐induced diabetic nephropathy rats. *Phytotherapy Research*.

[31] Shrestha S., Gyawali P., Shrestha R., Poudel B., Sigdel M. (2008). Serum urea and creatinine in diabetic and non-diabetic subjects. *Journal of Nepal Association for Medical Laboratory Sciences P*.

[32] Zhang L., Pang S., Deng B. (2012). High glucose induces renal mesangial cell proliferation and fibronectin expression through JNK/NF-*κ*B/NADPH oxidase/ROS pathway, which is inhibited by resveratrol. *The International Journal of Biochemistry & Cell Biology*.

[33] Lee H. B., Yu M.-R., Yang Y., Jiang Z., Ha H. (2003). Reactive oxygen species-regulated signaling pathways in diabetic nephropathy. *Journal of the American Society of Nephrology*.

[34] Levy Y., Zaltzberg H., Ben-Amotz A., Kanter Y., Aviram M. (1999). *β*-Carotene affects antioxidant status in non-insulin-dependent diabetes mellitus. *Pathophysiology*.

[35] Uddandrao V. V. S., Brahmanaidu P., Ravindarnaik R., Suresh P., Vadivukkarasi S., Saravanan G. (2019). Restorative potentiality of S-allylcysteine against diabetic nephropathy through attenuation of oxidative stress and inflammation in streptozotocin–nicotinamide-induced diabetic rats. *European Journal of Nutrition*.

[36] Saxena A. K., Srivastava P., Kale R. K., Baquer N. Z. (1993). Impaired antioxidant status in diabetic rat liver: effect of vanadate. *Biochemical Pharmacology*.

[37] Yan H., Harding J. J. (1997). Glycation-induced inactivation and loss of antigenicity of catalase and superoxide dismutase. *Biochemical Journal*.

[38] Ewis S. A., Abdel-Rahman M. S. (1995). Effect of metformin on glutathione and magnesium in normal and streptozotocin‐induced diabetic rats. *Journal of Applied Toxicology*.

[39] Tan S. M., de Haan J. B. (2014). Combating oxidative stress in diabetic complications with Nrf2 activators: how much is too much?. *Redox Report*.

[40] Gao P., Li L., Ji L. (2014). Nrf2 ameliorates diabetic nephropathy progression by transcriptional repression of TGF*β*1 through interactions with c-Jun and SP1. *Biochimica et Biophysica Acta (BBA) - Gene Regulatory Mechanisms*.

[41] Chen Q., Su Y., Ju Y., Ma K., Li W., Li W. (2018). Astragalosides IV protected the renal tubular epithelial cells from free fatty acids-induced injury by reducing oxidative stress and apoptosis. *Biomedicine & Pharmacotherapy*.

[42] Su S., Cao M., Wu G. (2018). Hordenine protects against hyperglycemia-associated renal complications in streptozotocin-induced diabetic mice. *Biomedicine & Pharmacotherapy*.

[43] Yang W.-J., Li Y.-R., Gao H. (2018). Protective effect of the ethanol extract from Ligusticum chuanxiong rhizome against streptozotocin -induced diabetic nephropathy in mice. *Journal of Ethnopharmacology*.

[44] Cui G., Chui Wah Luk S., Li R. A. (2015). Cytoprotection of baicalein against oxidative stress-induced cardiomyocytes injury through the Nrf2/Keap1 pathway. *Journal of Cardiovascular Pharmacology*.

[45] McMahon M., Thomas N., Itoh K., Yamamoto M., Hayes J. D. (2006). Dimerization of substrate adaptors can facilitate cullin-mediated ubiquitylation of proteins by a “tethering” mechanism. *Journal of Biological Chemistry*.

[46] Tong K. I., Katoh Y., Kusunoki H., Itoh K., Tanaka T., Yamamoto M. (2006). Keap1 recruits Neh2 through binding to ETGE and DLG motifs: characterization of the two-site molecular recognition model. *Molecular and Cellular Biology*.

[47] Chaitanya G. V., Alexander J. S., Babu P. P. (2010). PARP-1 cleavage fragments: signatures of cell-death proteases in neurodegeneration. *Cell Communication and Signaling*.

[48] Zhou L., An X.-F., Teng S.-C. (2012). Pretreatment with the total flavone glycosides of Flos Abelmoschus manihot and hyperoside prevents glomerular podocyte apoptosis in streptozotocin-induced diabetic nephropathy. *Journal of Medicinal Food*.

